# Preparation and Characterization of Antibacterial Films with Eggshell-Membrane Biopolymers Incorporated with Chitosan and Plant Extracts

**DOI:** 10.3390/polym14030383

**Published:** 2022-01-19

**Authors:** Brian Cameron Wooding Webb, Steven Rafferty, Andrew James Vreugdenhil

**Affiliations:** Department of Chemistry, Trent University, Peterborough, ON K9L 0G2, Canada; brianwebb@trentu.ca (B.C.W.W.); srafferty@trentu.ca (S.R.)

**Keywords:** biopolymer, eggshell membrane, chitosan, antibacterial, soluble eggshell membrane

## Abstract

A series of films containing chitosan (CS), eggshell membrane (ESM), soluble eggshell membrane (SEP), and plant extracts from *Thymus vulgaris* and *Origanum valgare* were prepared with varying concentrations and compositions. These novel films were characterized extensively with respect to film thickness and uniformity, solution absorption, degradation, microenvironmental pH, and antibacterial properties. All the films were flexible with appropriate mechanical stability. After 48 h of soaking in a lysozyme solution, all the films degraded 64 ± 4%, which would be expected to allow for the release of the plant extracts. The plant extracts on their own showed a pH of approximately 4, with the blended films having microenvironmental pHs from approximately 6.4–7.0, which would be expected to promote wound healing. A CS-ESM-SEP film with 5% of each plant extract inhibited almost all *E. coli* growth in liquid cultures and had no detriments to fluid absorption. Fluid absorption was approximately 100–150% by weight for all the films. The incorporation of SEP and plant extracts to a CS-ESM film provides a promising and novel method for the incorporation of SEP and antibacterial agents in a film with no detriment to wound fluid absorption or film degradation.

## 1. Introduction

The treatment of chronic wounds costs the United States $25 billion annually, affecting 6.5 million patients [1]. Wound dressings prepared from natural waste products may diminish these costs, improve patient outcomes, and introduce a more sustainably derived wound healing material. An ideal wound dressing must meet many requirements to be effective. The primary role of the dressing is to prevent infection during wound healing, but it is also advantageous that the dressing reduces wound necrosis, controls the moisture level of the wound, facilitates the removal of exudates, works to prevent infection, is cost-effective, and alleviates pain [2]. At the same time, it should be mechanically stable, biocompatible, and preferably biodegradable; given the scale of use, it should also be inexpensive to manufacture. Wound dressings based on blended biopolymer films are attractive, as they meet many of these requirements, and can be produced from renewable resources.

Chitosan (CS) is a natural amino polysaccharide of 2-acetamido-2-deoxy-β-D-glucose with β (1→4) linkages found in the shells of crustaceans and insects that has shown promise in the development of food packaging [3,4,5], and wound care products [6]. It has anti-infectious characteristics, good biocompatibility, hemostatic potential, low immunogenicity, and can be prepared in the form of blended films with the addition of glycerol as a plasticizer [7]. It is estimated that 60,000 to 80,000 tonnes of chitinous waste are produced from an average shell fish plant, making chitosan an abundant macromolecule for such applications [6].

Eggshell membrane (ESM), another biopolymer with potential applications in wound healing, consists of a water insoluble proteinaceous fibrous network found on the inside of eggshells [8]. This fibrous network contains collagens, amino acids, and other biologically active compounds [9,10]. The chemical composition of ESM has previously been extensively characterized in the literature [9,10]. ESM has a long history as a wound healing material by promoting the deposition of collagen, leading to thicker granulation tissue with faster wound healing and wound closure [11]. Previous work used chitosan and ESM with glycerol to prepare blended wound dressings and concluded that a film with 2% glycerol and 0.01 g mL^−1^ ESM and chitosan had the best potential as a wound-care product based on its absorption, degradation, antibacterial properties, and microenvironmental pH [12]. The outer layer fibers of ESM has calcium carbonate embedded in its organic matrix and this plays an integral role in providing structural integrity to CS-ESM blended films [12,13].

Like chitosan, ESM is also available in abundance; the egg-product industry is estimated to produce 5 million tonnes of eggshell by-product annually [12], approximately 10% of which is eggshell membrane [14]. On a per-company basis, the disposal of eggshell by-products costs between $25,000 to $100,000 annually [14]. Such costs could be diverted into revenue by using the available ESM waste product to produce a wound dressing, which could additionally decrease long-term wound treatment costs.

Soluble eggshell membrane protein (SEP) is a derivative of ESM that also has promising wound healing characteristics. The disulfide bond crosslinks present in ESM decrease its solubility and thus the bioavailability of soluble proteins and collagen within ESM [8]. SEP overcomes this solubility issue; through the reductive cleavage of crosslinking in ESM, the disulfide bonds are broken and SEP is formed, thus allowing for the greater solubility and availability of proteins and collagen to the surrounding environment [8]. SEP has been investigated for a variety of wound healing and tissue engineering applications. A dose response to SEP and gene expression has previously been detected using human dermal fibroblasts, which suggested an ECM environment is provided by SEP [15]. SEP has been electrospun with synthetic polymers and/or immobilized on the surface of electrospun scaffolds [16,17,18]. For example, SEP/poly (lactic-co-glycolic acid) (PLGA) electrospun membranes have been investigated for periodontal therapy, with good cell attachment, proliferation, and morphology [16].

Likely due to these alterations, SEP has been shown to have superior fibroblast adhesion properties than ESM, suggesting that it may be better suited as a wound healing material [19]. However, SEP does not retain the antibacterial properties of ESM [8]. Previous work has reported the preparation of a CS-SEP blended film with glycerol [20]. However, to our knowledge, the addition of SEP to a CS-ESM film (retaining the antibacterial properties of ESM) has yet to be explored. The combination of appropriate material availability due them being based on waste products, structural integrity, the strong fibroblast adhesion of SEP, and strong antibacterial properties, all suggest these films are best applied to treating chronic wounds, which still lack a suitable solution [2].

The antibacterial properties of a chronic wound dressing are crucial due to the cost of treatment and the detrimental affect chronic wound infections have on patient health [21]. Previously, CS films have been incorporated with antibacterial compounds such as minocycline [22] and silver nanoparticles [23,24,25]. Another approach employed to improve the antibacterial properties of biopolymer films is the incorporation of plant extracts [3,26,27,28], as they not only offer antibacterial properties but also antioxidants [28,29], and may have greater patient compliance due to the product having more compounds familiar with patients, as was previously demonstrated in the medical application of honey for wound care [30]. Plant oil extracts, such as the oil from thyme (*Thymus vulgaris*), have been incorporated into a chitosan film to explore its wound healing and antibacterial properties [28]. *Staphylococcus aureus* (*S. aureus*) and *Pseudomonas aeruginosa* (*P. aeruginosa*) are two of the most common causes of infection in chronic wounds [21]; their growth has been shown to be inhibited by the oils extracted from *Thymus vulgaris* (*T. vulgaris*) and *Origanum vulgare* (*O. vulgare*) [28], respectively, which were therefore chosen to be incorporated into the films in the current work.

In this work, the overall wound healing ability and antibacterial properties of the previously reported CS-ESM film [12] were improved upon with the addition of SEP and the incorporation of plant extracts (*T. vulgaris* and *O. vulgare*). To characterize and evaluate the prepared films’ viabilities as antibacterial wound dressings, the films’ thicknesses, mass, absorption of simulated wound fluid, microenvironment pH, degradation, Fourier-transform infrared spectroscopy (FT-IR) spectra, and antibacterial properties were all evaluated.

A moist wound bed is very important for wound healing as it provides nutrients; however, in chronic wounds, there can be an excess of this fluid leading to clinical complications [31]. It is therefore important that a chronic wound dressing act as a mediator, removing some fluid but still fostering an appropriate healing environment [31]. The pH of the microenvironment is critical to wound healing as it is thought to alter the activity of many reparative cells [32]. The degradation of the film will dictate the period over which the active compounds and antibacterial plant extracts will be eluted from the film. It is therefore critical that the films not immediately degrade nor hold their structural integrity for too many days. FT-IR was used to analyze the functional group changes caused by chitosan, SEP, and ESM, linking the films’ characterization results to their structure.

Based on this approach, the objective of this work was to prepare and characterize blended biopolymer films from ESM, SEP, chitosan, and plant extracts to achieve improved absorption, microenvironmental pH, and antibacterial properties than what has previously been reported. Providing the good structural integrity of the CS-ESM film, improved cell adhesion from SEP, and strong antibacterial properties of plant extracts are all integrated into the development of a novel film, this film should be superior for the treatment of chronic wounds when compared to the previously reported CS, ESM, and SEP blended films.

## 2. Materials and Methods

### 2.1. ESM Powder and Materials

Eggshells supplied by an industrial eggshell breaking facility (EggTech Ltd., Toronto, ON, Canada) were mechanically separated into eggshell and eggshell membrane, yielding wet eggshell membrane [33]. The wet membrane was dried at 50 °C overnight, powdered using the combination of a blender, a mortar and pestle, and passed through a sieve to obtain particles of 53-micron diameter or smaller. This powder was used as the ESM source for subsequent work described here. Acetic acid was purchased from ACP Chemicals (Montreal, QC, Canada) and used as received. Chitosan (low molecular weight (50,000–190,000 Da MW) deacylated chitin, poly(D-glucosamine)) was purchased from Sigma-Aldrich (Oakville, ON, Canada). The plant extracts were purchased from Aliksir (rue Saint-Joseph Est, QC, Canada), are USDA organic certified, and were extracted through steam distillation. *E. coli* strain K12 CW3747 was purchased from American Type Culture Collection (ATCC, Manassas, VA, United States). For all antibacterial testing lysogeny broth (LB) and agar (biological grade) were purchased from BioShop (Burlington, ON, Canada).

### 2.2. SEP Dissolution

To obtain SEP from ESM, a dissolution procedure was followed [34]. Dried, powdered ESM (0.03 g/mL) was added to 1.25 M aqueous 3-mercaptopropionic acid in the presence of 10% aqueous acetic acid. The solution was stirred for 12 h at 90 °C. The solution was then cooled to room temperature and centrifuged to remove insoluble material. Next, the pH of the solution was adjusted to 5 using 5 M NaOH, at which point a white precipitate of SEP formed in the solution. This white precipitate of SEP was collected by vacuum filtration, washed with methanol, and air dried.

### 2.3. Film Generation

The generation of all films was carried out following an adapted procedure [12]. A solution of 1 g chitosan powder per 100 mL of 1% aqueous acetic acid was prepared by heating the solution to 50 °C, with stirring, until the chitosan dissolved. Next, the intended mass of ESM, SEP, or both, was added to the chitosan solution and stirred until it had completely dispersed, at which point plant extracts were added if required. Lastly, 2% *v/v* glycerol was added. These steps were repeated with a series of ESM, SEP, and plant extract formulations, as indicated in Table 1.

From each formulation, 2.5 mL was pipetted and cast into 3.3 cm by 3.3 cm wells in a 15-well plate and left to dry at 37 °C. With the aim of allowing the film to provide the benefits of both ESM and SEP, the CS-ESM-SEP film was chosen to have the plant extracts incorporated.

### 2.4. Thickness and Uniformity

The thickness and uniformity of all generated films was determined by taking three thickness measurements of the films at different positions using electronic calipers. For each film, the three values were averaged, and the standard deviation was calculated.

### 2.5. Simulated Wound Fluid Absorption

To determine the absorption properties of the generated films, a previously published method was followed [12]. Simulated wound fluid (SWF) composed of 0.02 M CaCl_2_, 0.4 M NaCl, 0.08 M tris methylamine, and 2% *w/v* of bovine serum albumin (BSA) was prepared. The films were weighed (W_0_) and then immersed in the SWF for 150 min, with the mass being recorded (W_s_) every 15 min. When weighing the films, any excess fluid that appeared on the film was blotted off. The following formula was then used to calculate the wound fluid absorption (WFA) of the films.

WFA (%) = (W_s_ − W_0_)/W_0_ × 100% (1)

### 2.6. Microenvironment pH

The pH of the microenvironment created by the generated films was determined following a previously established method [12]. Either a piece of film (12 mm × 12 mm) or 0.1 mL of each plant extract was placed in 4 mL of 0.9% saline solution (normal saline) at 25 °C for 24 h. The films were removed from the saline solution and the pH of the solution was measured. This procedure was carried out in triplicate.

### 2.7. Fourier-Transform Infrared Spectroscopy (FT-IR)

FT-IR spectra from 500 cm^−1^ to 3500 cm^−1^ of the prepared films were obtained using a Thermo Fisher Scientific (Spectrometer, Markham, Thermo Fisher Scientific, ON, Canada) Nicolet 380 FT-IR using a single bounce ZnSe ATR accessory (MIRacle ATR, Pike Technologies, Fitchburg, WI, United States). Typically, 32 scans were co-added to obtain appropriate signal to noise ratios. A background of the empty crystal was obtained prior to sample analysis using the same parameters.

### 2.8. Degradation

To assess the degradation of the prepared films, an adapted procedure from was followed [35]. Pieces of film (12 mm × 12 mm) were weighed (W_i_) and soaked in 1 mg mL^−1^ lysozyme/PBS solution for 48 and 77 h at 37 °C. The films were then frozen and freeze-dried for 27 h. The freeze-dried films were weighed (W_t_) and the relative degradation (RD) was calculated using the following equation.

RD (%) = (W_i_ − W_t_)/W_i_ × 100% (2)

### 2.9. Antibacterial Assessment

All utensils were immersed in ethanol followed by flame sterilization; a sterile work environment was used by wiping the lab bench area with 70% ethanol and working in the vicinity of a Bunsen burner. Luria broth (LB) and LB agar media were autoclaved for 45 min at 121 °C on a liquid cycle. All sample films were 0.25 inches in diameter and were sterilized by exposing them to UV-light from a Fotodyne Fotoprep I 3500 UV light box (UV Transilluminator, Fotodyne, Hartland, WI, United States) for 5 min on the analytical setting.

#### 2.9.1. Plate Cultures

Starter cultures of *E. coli* strain K12 were prepared by inoculating 2 mL of LB media and grown in a shaker-incubator for 16–20 h at 37 °C and 250 rpm. A serial dilution was then performed, with the overnight culture ultimately diluted 10,000-fold. Of this diluted sample, 100 µL was used to inoculate each plate. The sterilized films were put in SWF for 50 min, the SWF having been centrifuged in a microcentrifuge at 13,000× *g* to sterilize the liquid, removing any insoluble material. The soaked films were then placed on inoculated plates and were incubated for 21 h at 37 °C. A positive (with *E. coli*) and negative (without *E. coli*) control were used. The zone of inhibition on the plate around each film was measured with a ruler.

#### 2.9.2. Liquid Cultures

As a more sensitive antibacterial testing method, the inhibition of liquid LB cultures of *E. coli* was measured. A starter culture with 2 mL of LB was inoculated with K12 *E. coli* and grown overnight for 20 h at 37 °C and 250 rpm. From this, a serial dilution in LB liquid media was performed, diluting the sample 100,000-fold. Eight culture tubes, each containing a different sterilized film, were inoculated with 2 mL of the diluted *E. coli* culture. In addition, a positive control of diluted *E. coli* culture and no film, and a negative control of 2 mL sterile LB alone, were prepared. This was completed in triplicate for a total of 30 culture tubes. The cultures were incubated for 19 h at 37 °C and 250 rpm. The optical density (OD) was then measured at 600 nm using a Shimadzu UV-160 spectrophotometer (UV Spectrophotometer, Shimadzu, Columbia, MD, United States).

## 3. Results

### 3.1. Film Preparation and Structural Integrity

**Figure 1:**
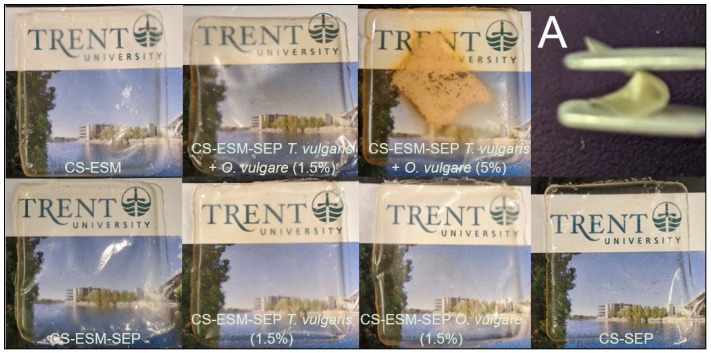
Images of all prepared films. (**A**) A piece of the CS-ESM-SEP film being bent by forceps.

The blended CS-SEP and CS-ESM-SEP films were successfully generated with the incorporation of plant extracts, as shown in Figure 1.

In preparing the films, the solubility of SEP was poor in 1% acetic acid; however, given sufficient stirring time, all particles became dispersed. The incorporation of the plant extracts did cause a noticeable alteration in coloration and surface morphology due to the insolubility and dispersion of the plant extracts within the films. This was most noticeable in the film with 5% of each plant extract. Mechanical integrity was not significantly compromised in any of the films. All the films had very similar structural properties to that displayed in Figure 1A. All the films had a thickness in the range of 0.16 to 0.09 mm, with masses ranging from 0.12 g to 0.13 g. No obvious trends or consequential differences in thickness or mass were observed among the different films. The films had no large standard deviations in thickness, nor relevant differences in mass, indicating good film uniformity. The films with 1.5% and 5% of each plant extract did have the greatest standard deviation (±0.04 mm) in thickness, with this likely being due to the presence of plant extracts within the films; however, this variation did not appear to compromise the films’ structural integrity.

### 3.2. Absorption

**Figure 2:**
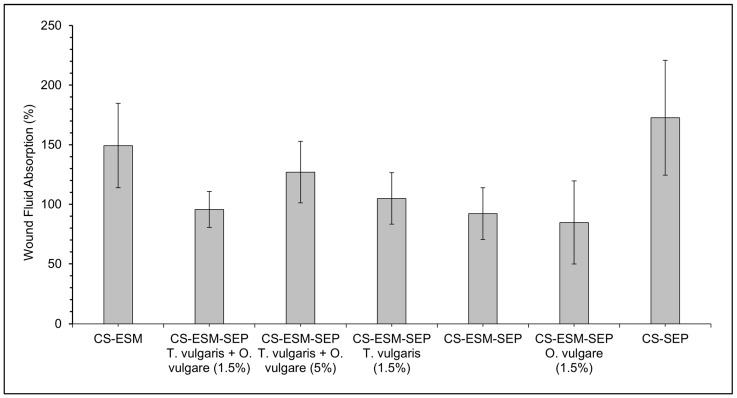
The wound fluid absorption percent of the prepared films tested every 15 min over 150 min.

The wound fluid absorption of a wound dressing is critical for determining the film’s ability to mediate the fluid and nutritional environment within a wound. All the CS-ESM-SEP films, with or without plant extracts, showed similar wound fluid absorption, with the CS-ESM-SEP film having only a slight decrease in wound fluid absorption, as shown in Figure 2.

### 3.3. FT-IR Analysis

**Figure 3:**
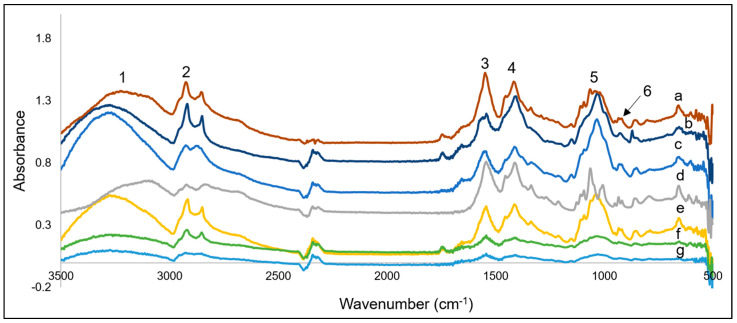
FT-IR spectra of films: (**a**) CS-ESM-SEP *T. vulgaris* + *O. vulgare* (5%), (**b**) CSSEP, (**c**) CS-ESM-SEP, (**d**) CS-ESM, (**e**) CS-ESM-SEP *O. vulgare* (1.5%), (**f**) CS-ESM-SEP *T. vulgaris* (1.5%), and (**g**) CS-ESM-SEP *T. vulgaris* + *O. vulgare* (1.5%).

FT-IR spectra of all the prepared films were acquired to illuminate changes or similarities in functionality between the prepared films, as shown in Figure 3.

The hydrophilic, and therefore absorption, properties of the films are largely due to the presence of O-H and N-H functional groups. Between 3500–3000 cm^−1^ (indicated by 1 in Figure 3) both O-H and N-H stretching were detected and overlapping [7]. These amine and hydroxyl groups are present in chitosan and responsible for hydrogen bonding with both other chitosan molecules and glycerol contributing to the structural integrity of the films [22]. The double peak between ~3000–2800 cm^−1^ (indicated by 2 in Figure 3) is indictive of CH_2_, specifically the asymmetric mode [28]. The absorption around 1600 cm^−1^ (indicated by 3 in Figure 3) is characteristic of C=O stretching, and is overlapped with N-H bending [7]. This is characteristic of N-acetylglucosamine, indicating the chitosan used was not fully deacetylated, as expected [7,36,37,38]. Furthermore, SEP also contributes to the amide-I band around 1600 cm^−1^ [39]. The peak at ~1400 cm^−1^ (indicated by 3 in Figure 3) represents the -CH_3_ symmetrical deformation [7]. The peak at ~1050 cm^−1^ (indicated by 5 in Figure 3) is due to C-O stretching [7]. The peak at ~900 (indicated by 6 in Figure 3) corresponds to the *β*-pyranoside bond present in chitosan [7].

### 3.4. Microenvironmental pH

**Figure 4:**
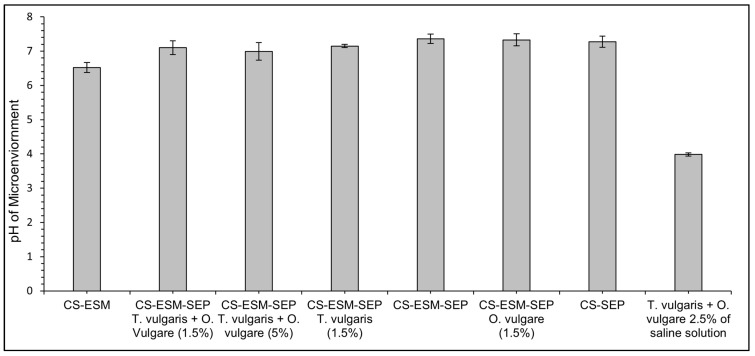
The microenvironmental pH created by the films and plant extracts after 24 h in normal saline solution at 25 °C.

The pH of a wound greatly affects the wound healing process, with a lower pH promoting angiogenesis, increasing macrophage and fibroblast activity, and increasing oxygen within the wound, resulting in promoted healing [32]. The pH of the microenvironment created by the prepared films did not vary significantly from the CS-ESM films, demonstrating a slightly lower pH microenvironment by approximately 0.5 pH units, as shown in Figure 4, with the pH ranging from 6.5 to 7 for various formulations.

### 3.5. Degradation

The degradation of these films provides insight into the period over which the antibacterial plant extracts and proteins will be released from the films. After 48 h of soaking in a lysozyme solution and being freeze dried, all the films had similar relative degradation (RD), with the average weight loss being 64 ± 4%.

After 76 h of soaking in lysozyme solution, all the films degraded beyond what could be removed with forceps due to either their delicate state or being in too many pieces.

### 3.6. Antibacterial Testing

#### 3.6.1. Plate Cultures

For the generated films to be considered as adequate chronic wound dressings, they must have antibacterial properties in addition to promoting the wound healing process [31,40]. Plate cultures show that the CS-ESM-SEP *T. vulgaris* and *O. vulgare* (5%) film provides a 1.3 cm zone of inhibition against the growth of *E. coli*, demonstrating the films’ antibacterial properties when plant extracts are included. No other films displayed a zone of inhibition on plate cultures. Liquid cultures were subsequently used to provide a more sensitive testing method.

#### 3.6.2. Liquid Cultures

**Figure 5:**
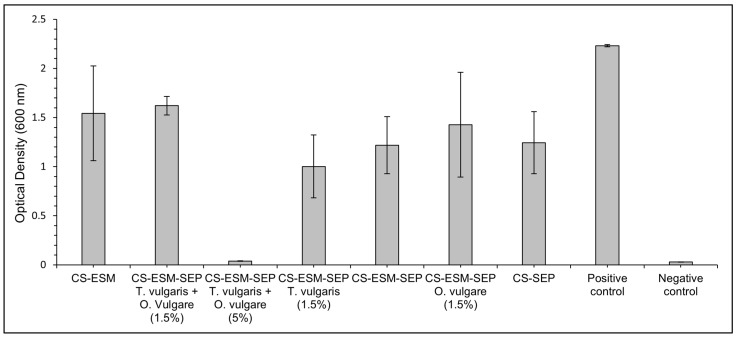
Optical density of liquid culture samples after 19 h of incubation. The positive control was inoculated, whereas the negative control was not.

The liquid *E. coli* cultures revealed that after 19 h of incubation, all the films provided an antibacterial affect, showing lower optical density (OD) values than the positive control, as shown in Figure 5.

The CS-ESM-SEP *T. vulgaris* and *O. vulgare* (5%) film showed exceptional antibacterial properties, completely preventing the growth of the *E. coli*, having an OD of 0.04 ± 0.00, compared to the OD of the positive control, which was 2.23 ± 0.01.

## 4. Discussion

The observed flexibility of the films is likely due to the glycerol element, which is a common additive used to blend biopolymers films, as it has been shown to improve the mechanical properties chitosan films and stabilize them from dissolution [41]. While the glycerol provides appropriate mechanical properties for molding to wounds, the addition of ESM and SEP reinforce the polymer network, resulting in a film not easily dissolved in PBS [42].

Similarly to what has been previously concluded with a chitosan glycerol film, the added compounds in this study appeared to blend with inter-/intra-hydrogen bonding [7]. If a chemical reaction such as covalent bonding was occurring between the incorporated compounds, a greater difference in functionality would be expected in the FT-IR results.

The absorption properties of a dressing are a function of its hydrophilic properties [2]. Compared to raw ESM, SEP has fewer disulfide bonds between cysteine residues and consequentially greater hydrophilicity [19]. Thus, it was expected that the films with SEP would have a greater SWF absorption. The CS-SEP film did show greater absorption, which provides support for the idea that the greater hydrophilicity of SEP may be responsible for increasing the films absorption of SWF.

Surprisingly, despite the percentage of ESM or SEP remaining constant, the CS-ESM-SEP films showed a slight decrease in wound fluid absorption compared to the CS-ESM and CS-SEP films. Considering that the only difference between SEP and ESM is the lack of disulfide bonds, it is suggested that, once blended, the lack of disulfide bonds, and thus the improved solubility of SEP, may allow for greater intermolecular H-bonding between SEP, ESM, and glycerol, resulting in a film which can therefore participate less in H-bonding with the surrounding fluid; however, due to the decrease in WFA only being slight, it would likely have no impact on the film’s clinical applications.

The mediation of excess wound fluid plays a role in nutrient delivery and can aid in reducing the chances of complications in healing [31]. All the prepared films absorbed ~1–1.5 times their weight in fluid, indicating that they would remove exudate fluid and mediate the moisture and nutrients within the wound bed. In turn, this allows for greater fibroblast adhesion and proliferation, and accelerated wound recovery [12]. The CS-ESM base film for this work had glycerol incorporated into it, which increases the hydrophilicity of the dressing and is largely responsible for the dressing’s fluid absorption [12]. The amine and hydroxyl functionality found in chitosan [43], and the hydrophilicity of SEP, meanwhile, provide additional absorption properties [19]. The CS-ESM film had an expected WFA of 200 ± 25% according to previously published research, and our results are consistent with this, showing a WFA of 180% for the same material, which indicates that our method of absorption testing was reliable [12].

The incorporation of essential oils to biopolymer blended films typically comes at the cost of a decreased degree of absorption due to the hydrophobic properties of essential oils [27]; however, when we compared the CS-ESM-SEP film with no plant extracts to the CS-ESM-SEP *T. vulgaris* + *O. vulgare* (5%) film, we observed an increase in absorption. This may be due to the incorporation of plant extracts decreasing the availability of hydrogen groups to interact between CS, ESM, SEP, and glycerol, thus allowing for greater H-bonding to occur with the SWF. Most importantly, the CS-ESM-SEP film with 5% of each extract had no significant detrimental impact on absorption compared to the CS-ESM film, indicating that the addition of SEP and antibacterial plant extracts comes at no sacrifice to fluid absorption, an issue that has been previously noted with similar biopolymer films [27].

Based on previous work, the CS-ESM had an expected microenvironmental pH of ~5.9, slightly lower than the pH of ~6.5 found in the current study [12]. The incorporation of SEP does appear to increase the pH microenvironment. The slightly larger standard deviation in the CS-ESM-SEP film with 5% *T. vulgaris* and *O. vulgare* incorporated might be due to the inconsistent distribution of the plant extracts within the film. It is expected that each 5% extract film as a whole would provide the same pH due to the same volume of plant extracts being present and eventually being distributed within the wound bed’s fluid. However, future work could employ a method in which air is blown over the film during the drying process to increase the dispersion of plant extracts [27].

Although the plant extracts alone in saline were shown to result in a much lower pH (~4), this was not seen in the films with plant extracts incorporated (Figure 4). This suggests the films provide some beneficial buffering capacity. The buffering capacity of CS and its derivatives have been discussed before, mainly due to their potential applications as delivery vehicles [44,45,46]. Furthermore, the buffering capacity of chitosan has been shown to be higher in pH ranges from 4.5–7 than other pH ranges [46], which is between the pH observed with the plant extracts. The buffering capacity of chitosan is largely provided by the primary amine [47]. It is interesting that the microenvironmental pH appears to increase with the addition of SEP (and lack of ESM). This is likely due to the increased buffering capacity provided by the thiol groups formed when the disulfide bonds of ESM are broken to yield SEP.

When evaluating the degradation of biopolymer films, it is important to distinguish between degradation and dissolution. Previously, CS-ESM films have shown little to no dissolution when submerged in PBS, whereas chitosan films have been shown to dissolute in the same conditions [12]. This is explained by the release of calcium ions from ESM in the acidic cast environment leading to electrostatic attraction between the chitosan and calcium, producing a reinforced network [12,13]. Additionally, the ESM, SEP, and plant extracts may be functioning as physical fillers providing further reinforcement [42]. The degradation results reported here, combined with previous reports, all suggest that it is likely that the CS, ESM, and SEP provide the structural integrity to these novel films; however, when interpreting the data, it is important to appreciate that lysozyme is expected to mainly degrade chitosan through the hydrolytic cleavage of glycosidic bonds between glucosamine, N-acetyl-glucosamine, and themselves [35,48]. Thus, it is supported that in vivo the incorporated plant extracts would be released, decreasing the pH more than other blended biopolymer-based films, providing superior wound healing [32]. Furthermore, the ESM, chitosan, and SEP would also become available in the wound bed, promoting wound healing in a timely manner.

In this study, all the films had been freeze dried before being weighed rather than using wetted masses. One advantage of this is that the degradation of the films’ polymer chains could occur in such a way that the absorption of the films is consequentially affected. For example, it is the glycosidic bonds which are cleaved by lysosome, making chitosan particularly venerable to degradation [35,48]. It would be expected that the swelling properties of a blended biopolymer film has a functional relationship with the presence of chitosan. Thus, it if is mainly chitosan being degraded and being removed from the polymer film, it is expected that the films’ swelling would be directly affected. Therefore, by freeze drying the films rather than using the wet mass, potentially confounding factors are removed. This supports the validity of this testing method compared to previously reported methods which did not account for differences in absorptivity between the films [12].

The microenvironmental pH results, taken with the degradation results, support the idea that that the films would facilitate the timely release of the antibacterial plant extracts or any other additives, providing continuous protection against infection and a decrease in microenvironmental pH. Other films, such as a PEG-cross-linked chitosan film, have relatively slow degradation (21% after 24 days in a lysosome solution) [35]. The difference in degradation is likely a reflection of the PEG-cross-linked chitosan film being covalently crosslinked, greatly increasing the films stability. This relatively slow degradation would be much slower than what would be required for the affective release of plant extracts and biopolymers in a timely manner, suggesting a blended non-covalently crosslinked film is likely more effective for the delivery of plant extracts and biopolymers to the wound bed.

In previous work, improved film integrity was achieved with the incorporation of ESM to a chitosan film [12], which appears to have not been compromised by the addition of SEP or plant extracts. A previously reported CS-SEP film was determined to disperse in water after only one hour, which provides challenges for clinical translation as the product would need to be constantly replaced [20]. Therefore, the films prepared in this study may be more suited for clinical applications due to improved degradation properties and film stability, while still delivering the same amount of SEP, just over a more practical time frame.

Due to SEP not retaining the same antibacterial properties as ESM [8], it was expected that the CS-SEP film would have significantly reduced antibacterial activity than the films with ESM. However, this was not the case, as the CS-ESM, CS-ESM-SEP, and CS-SEP films had no major differences in antibacterial activity. This is most likely due to 0.01 or 0.005 g/mL ESM and/or SEP not being a high enough concentration to allow for the compounds’ different antibacterial capabilities to be detectable in these results.

One study looking at the antibacterial affects of plant extracts found that *T. vulgaris* and *O. vulgare* provided a ~32.4 mm and ~29.5 mm zone of inhibition against *E. coli*, respectively [49]. Following the same procedure using *T. vulgaris* and *O. vulgare*, a zone of inhibition greater than 90 mm was found against both *Staphylococcus aureus* and *Pseudomonas aeruginosa* [49], two of the most common types of chronic wound infections [21]. These results therefore support the expectation that the CS-ESM-SEP *T. vulgaris* and *O. vulgare* (5%) film would have exceptional antibacterial properties against chronic wound infections. The fact that the microenvironmental pH was relatively similar in all the films when soaked in saline suggests that the antibacterial activity was not a consequence of pH differences in the liquid cultures.

Currently, the efficacy of many other available treatment options for chronic wounds, such as grafting or seeded scaffolds, is diminished due to high rates of infection [40]. Therefore, it was very valuable that the prepared films have strong antibacterial activity. Although other approaches, such as the incorporation of compounds like minocycline [22] and silver nanoparticles [23,24,25] into CS films, has been explored, plant extracts are unique in that they not only provide antibacterial properties but also decrease pH, provide antioxidants [28,29], and may have greater patient compliance due to the product having more patient-familiar compounds [30].

## 5. Conclusions

A new type of blended biopolymer film containing chitosan, ESM, SEP, and plant extracts was prepared. Based on the results, the film containing CS-ESM-SEP *T. vulgaris* and *O. vulgare* (5%) would be the best suited for an antibacterial chronic wound dressing, due to the presence of chitosan, ESM, SEP, and plant extracts. The successful incorporation of SEP and plant extracts into a CS-ESM blended film with no significant detriments to physical characteristics, microenvironmental pH (ranging from ~6.5–7.0 pH units), fluid absorption, and degradation (average weight loss of 64% ± 4% after 48 h) has been accomplished, allowing for the incorporation of SEP and plant extracts into an appropriately degrading antibacterial film. Thus, some determinants that are reported with generating blended films with plant extracts have been overcome or minimized. Furthermore, the reported films are mainly comprised of waste products which may have positive economic and environmental impacts. Compared to what is currently available, the results suggest that a potentially superior chronic wound dressing has been generated which has strong antibacterial properties and allows for the delivery of SEP.

## Figures and Tables

**Table 1:** Sample formulation parameters: a dash indicates the material was not used for that formulation.

	Chitosan Powder (g mL^−1^)	ESM (g mL^−1^)	SEP (g mL^−1^)	Glycerol (*v/v*)	*T. vulgaris* Oil (*v/v*)	*O. vulgare* Oil (*v/v*)
CS-ESM	0.01	0.01	-	2%	-	-
CS-ESM-SEP	0.01	0.005	0.005	2%	-	-
CS-SEP	0.01	-	0.01	2%	-	-
CS-ESM-SEP *T. vulgaris*	0.01	0.005	0.005	2%	1.5%	-
CS-ESM-SEP *O. vulgare*	0.01	0.005	0.005	2%	-	1.5%
CS-ESM-SEP *T. vulgaris* + *O. vulgare* (1.5%)	0.01	0.005	0.005	2%	1.5%	1.5%
CS-ESM-SEP *T. vulgaris* + *O. vulgare* (5%)	0.01	0.005	0.005	2%	5%	5%

## Data Availability

The data presented in this study are available on request from the corresponding author.

## References

[1] Sen C.K., Gordillo G.M., Roy S., Kirsner R., Lambert L., Hunt T.K., Gottrup F., Gurtner G.C., Longaker M.T. (2009). Human Skin Wounds: A Major and Snowballing Threat to Public Health and the Economy. Wound Repair Regen..

[2] Ghomi E.R., Khalili S., Khorasani S.N., Neisiany R.E., Ramakrishna S. (2019). Wound Dressings: Current Advances and Future Directions. J. Appl. Polym. Sci..

[3] Kamkar A., Molaee-aghaee E., Khanjari A., Akhondzadeh-basti A., Noudoost B., Shariatifar N., Alizadeh Sani M., Soleimani M. (2021). Nanocomposite Active Packaging Based on Chitosan Biopolymer Loaded with Nano-Liposomal Essential Oil: Its Characterizations and Effects on Microbial, and Chemical Properties of Refrigerated Chicken Breast Fillet. Int. J. Food Microbiol..

[4] Wang C., Chang T., Dong S., Zhang D., Ma C., Chen S., Li H. (2020). Biopolymer Films Based on Chitosan/Potato Protein/Linseed Oil/ZnO NPs to Maintain the Storage Quality of Raw Meat. Food Chem..

[5] Souza V.G.L., Pires J.R.A., Rodrigues C., Coelhoso I.M., Fernando A.L. (2020). Chitosan Composites in Packaging Industry-Current Trends and Future Challenges. Polymers.

[6] Kumar M.N.V.R. (2000). A Review of Chitin and Chitosan Applications. React. Funct. Polym..

[7] Ma Y., Xin L., Tan H., Fan M., Li J., Jia Y., Ling Z., Chen Y., Hu X. (2017). Chitosan Membrane Dressings Toughened by Glycerol to Load Antibacterial Drugs for Wound Healing. Mater. Sci. Eng. C.

[8] Sah M.K., Rath S.N. (2016). Soluble Eggshell Membrane: A Natural Protein to Improve the Properties of Biomaterials Used for Tissue Engineering Applications. Mater. Sci. Eng. C.

[9] Tsai W.T., Yang J.M., Lai C.W., Cheng Y.H., Lin C.C., Yeh C.W. (2006). Characterization and Adsorption Properties of Eggshells and Eggshell Membrane. Bioresour. Technol..

[10] Nakano T., Ikawa N.I., Ozimek L. (2003). Chemical Composition of Chicken Eggshell and Shell Membranes. Poult. Sci..

[11] Ahmed T.A.E., Suso H., Maqbool A., Hincke M.T. (2019). Processed Eggshell Membrane Powder: Bioinspiration for an Innovative Wound Healing Product. Mater. Sci. Eng. C.

[12] Li X., Ma M., Uk D., Huang X. (2019). Preparation and Characterization of Novel Eggshell Membrane-Chitosan Blend Films for Potential Wound-Care Dressing: From Waste to Medicinal Products. Int. J. Biol. Macromol..

[13] Hincke M.T., Gautron J., Panheleux M., Garcia-Ruiz J., McKee M.D., Nys Y. (2000). Identification and Localization of Lysozyme as a Component of Eggshell Membranes and Eggshell Matrix. Matrix Biol..

[14] MacNeil H.J. (2006). Method and Apparatus for Separating a Protein Membrane and Shell Material in Waste Egg Shells. Patent.

[15] Ohto-fujita E., Konno T., Shimizu M., Ishihara K., Sugitate T., Niyake J., Yoshimura K., Taniwaki K., Sakurai T., Hasebe Y. (2011). Hydrolyzed Eggshell Membrane Immobilized on Phosphorylcholine Polymer Supplies Extracellular Matrix Environment for Human Dermal Fibroblasts. Cell Tissue Res..

[16] Jia J., Liu G., Guo Z., Yu J., Duan Y. (2012). Preparation and Characterization of Soluble Eggshell Membrane Protein/PLGA Electrospun Nanofibers for Guided Tissue Regeneration Membrane. J. Nanomater..

[17] Jia J., Duan Y., Yu J., Lu J. (2007). Preparation and Immobilization of Soluble Eggshell Membrane Protein on the Electrospun Nanofibers to Enhance Cell Adhesion and Growth. J. Biomed. Mater. Res. Part A.

[18] Yi F., Guo Z., Hu P., Fang Z., Yu J., Li Q. (2004). Mimetics of Eggshell Membrane Protein Fibers by Electrospinning. Macromol. Rapid Commun..

[19] Yi F., Guo Z., Zhang L., Yu J., Li Q. (2004). Soluble Eggshell Membrane Protein: Preparation, Characterization and Biocompatibility. Biomaterials.

[20] Qi Q., Lu J., Guo Z., Yu J. (2009). Preparation and Characterization of Soluble Eggshell Membrane Protein/Chitosan Blend Films. Chin. J. Polym. Sci..

[21] Serra R., Grande R., Amato B., Butrico L., Rossi A., Settimio Francesco U., Caroleo B., Gallelli L., de Franciscis S. (2015). Chronic Wound Infections: The Role of Pseudomonas Aeruginosa and Staphylococcus Aureus. Expert Rev. Anti. Infect. Ther..

[22] Zoghi N., Fouani M.H., Bagheri H., Nikkhah M., Asadi N. (2021). Characterization of Minocycline Loaded Chitosan/Polyethylene Glycol/Glycerol Blend Films as Antibacterial Wound Dressings. J. Appl. Polym. Sci..

[23] Xiao X., Tong Z., Liao J., Wang T. (2020). High-Efficient and Synergetic Antibacterial Nanocomposite Hydrogel with Quaternized Chitosan/Ag Nanoparticles Prepared by One-Pot UV Photochemical Synthesis. Biopolymers.

[24] Thomas V., Yallapu M.M., Sreedhar B., Bajpai S.K. (2009). Fabrication, Characterization of Chitosan/Nanosilver Film and Its Potential Antibacterial Application. J. Biomater. Polym. Ed..

[25] Kadam D., Momin B., Palamthodi S., Lele S.S. (2019). Physicochemical and Functional Properties of Chitosan-Based Nano- Composite Films Incorporated with Biogenic Silver Nanoparticles. Carbohydr. Polym..

[26] Wang L., Liu F., Jiang Y., Chai Z., Li P., Cheng Y., Jing H., Leng X. (2011). Synergistic Antimicrobial Activities of Natural Essential Oils with Chitosan Films. J. Agric. Food Chem..

[27] Yeddes W., Djebali K., Aidi Wannes W., Horchani-Naifer K., Hammami M., Younes I., Saidani Tounsi M. (2020). Gelatin-Chitosan-Pectin Films Incorporated with Rosemary Essential Oil: Optimized Formulation Using Mixture Design and Response Surface Methodology. Int. J. Biol. Macromol..

[28] Altiok D., Altiok E., Tihminlioglu F. (2010). Physical, Antibacterial and Antioxidant Properties of Chitosan Films Incorporated with Thyme Oil for Potential Wound Healing Applications. J Mater. Sci. Mater. Med..

[29] Tepe B., Daferera D., Sokmen M., Polissiou M., Sokmen A. (2004). In Vitro Antimicrobial and Antioxidant Activities of the Essential Oils and Various Extracts of Thymus Eigii, M. Zphary et P.H. Davis. J. Agric. Food Chem..

[30] Simon A., Traynor K., Santos K., Blaser G., Bode U., Molan P. (2009). Medical Honey for Wound Care—Still the ‘Latest Resort’?. Ecam.

[31] Boateng J.S., Matthews K.H., Stevens H.N.E., Eccleston G.M. (2008). Wound Healing Dressings and Drug Delivery Systems: A Review. J. Pharm. Sci..

[32] Gethin G. (2007). The Significance of Surface PH in Chronic Wounds. Wounds UK.

[33] Toman M., Kwinter S., Vreugdenhil A. (2010). Seperation of calcium carbonate eggshells from organic membrane. WO Patent.

[34] Yi F., Yu J., Guo Z., Zhang L., Li Q. (2003). Natural Bioactive Material: A Preparation of Soluble Eggshell Membrane Protein. Macromol. Biosci..

[35] Tanuma H., Saito T., Nishikawa K., Dong T., Yazawa K., Inoue Y. (2010). Preparation and Characterization of PEG-Cross-Linked Chitosan Hydrogel Films with Controllable Swelling and Enzymatic Degradation Behavior. Carbohydr. Polym..

[36] Balau L., Lisa G., Popa M.I., Tura V., Melnig V. (2004). Physico—Chemical Properties of Chitosan Films. Cent. Eur. J. Chem..

[37] Lawrie G., Keen I., Drew B., Chandler-temple A., Rintoul L., Fredericks P., Grøndahl L. (2007). Interactions between Alginate and Chitosan Biopolymers Characterized Using FTIR and XPS. Biomacromolecules.

[38] Nunthanid J., Puttipipatkhachorn S., Yamamoto K., Garnet E., Nunthanid J., Puttipipatkhachorn S. (2001). Physical Properties and Molecular Behavior of Chitosan Films Physical Properties and Molecular Behavior of Chitosan Films. Drug Dev. Ind. Pharm..

[39] Yi F., Lu J., Guo Z., Yu J. (2006). Mechanical Properties and Biocompatibility of Soluble Eggshell Membrane Protein/Poly (Vinyl Alcohol) Blend Films. J. Biomater. Sci. Polym. Edn..

[40] Harding K.G., Morris H.L., Patel G.K. (2002). Healing Chronic Wounds. BMJ.

[41] Wang S., Jing Y. (2017). Study on the Barrier Properties of Glycerol to Chitosan Coating Layer. Mater. Lett..

[42] Shankar S., Rhim J.W. (2018). Preparation of Sulfur Nanoparticle-Incorporated Antimicrobial Chitosan Films. Food Hydrocoll..

[43] Auta M., Hameed B.H. (2014). Chitosan—Clay Composite as Highly Effective and Low-Cost Adsorbent for Batch and Fixed-Bed Adsorption of Methylene Blue. Chem. Eng. J..

[44] Riva R., Ragelle H., Des Rieux A., Duhem N., Jérôme C., Préat V. (2011). Chitosan and Chitosan Derivatives in Drug Delivery and Tissue Engineering. Adv. Polym. Sci..

[45] Lu B., Wang C.F., Wu D.Q., Li C., Zhang X.Z., Zhuo R.X. (2009). Chitosan Based Oligoamine Polymers: Synthesis, Characterization, and Gene Delivery. J. Control. Release.

[46] Richard I., Thibault M., de Crescenzo G., Buschmann M.D., Lavertu M. (2013). Ionization Behavior of Chitosan and Chitosan−DNA Polyplexes Indicate That Chitosan Has a Similar Capability to Induce a ProtonSponge Effect as PEI. Biomacromolecules.

[47] Moreira C., Oliveira H., Pires L.R., Simões S., Barbosa M.A., Pêgo A.P. (2009). Improving Chitosan-Mediated Gene Transfer by the Introduction of Intracellular Buffering Moieties into the Chitosan Backbone. Acta Biomater..

[48] Loncarevic A., Ivankovic M., Rogina A. (2017). Lysozyme-Induced Degradation of Chitosan: The Characterisation of Degraded Chitosan Scaffolds. J. Tissue Repair Regen..

[49] Dorman H.J.D., Deans S.G. (2000). Antimicrobial Agents from Plants: Antibacterial Activity of Plant Volatile Oils. J. Appl. Microbiol..

